# Endoscopic fully covered self-expanding metallic stent placement combined with a novel over the-scope clip for the closure of a complex esophagomediastinal fistula

**DOI:** 10.1055/a-2811-5664

**Published:** 2026-03-05

**Authors:** Qi Gong, Jinxin Chen, Fucheng Zhang, Min Li, Fuyan Wang, Zhi Wei

**Affiliations:** 1 611300 Department of Gastroenterology, Shandong Second Provincial General Hospital, Jinan, China

**Fig. 1:**
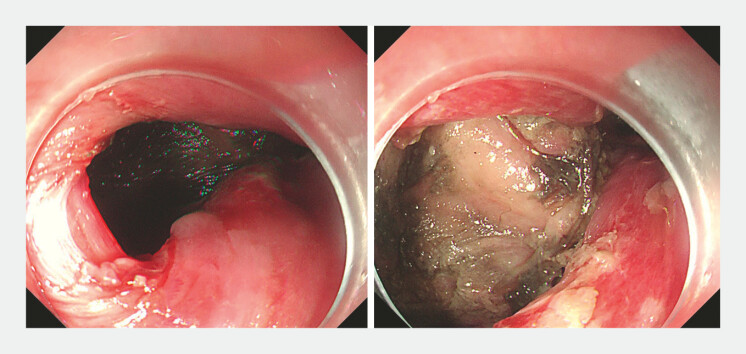
EGD showed an esophageal perforation (20 mm × 20 mm) at the lower esophagus site and extraesophageal leakage into the mediastinum. EGD, esophagogastroduodenoscopy.

**Fig. 2:**
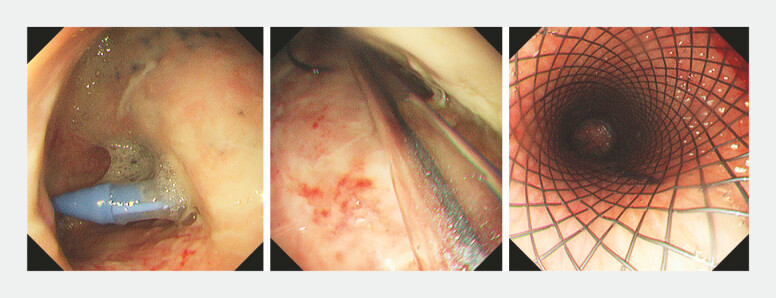
Endoscopic mediastinal lavage with catheter placement and deployment of a recyclable covered metal stent were performed.

A 53-year-old man was admitted to our hospital after spontaneous esophageal perforations caused by severe nausea and vomiting. Esophagogastroduodenoscopy showed an esophageal perforation (20 mm × 20 mm) at the lower esophagus site and extraesophageal leakage into the mediastinum ( Fig. 1 ). Because of severe mediastinal infection, the thoracic surgeon decided to perform conservative treatment rather than immediate surgery. Intravenous antibiotics and proton pump inhibitors were administered immediately. Four weeks later, endoscopic mediastinal lavage with catheter placement and deployment of a recyclable covered metal stent were performed ( Fig. 2 ). Enteral nutrition was started using an indwelling gastric tube and treatment with intravenous nutrition.

**Fig. 3:**
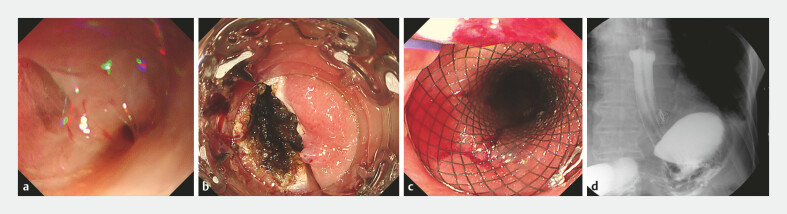
**a** Gastroscopy revealed an approximately 6 mm fistula in the lower esophagus. **b** The mucosa around the fistula was cauterized by APC. **c** Deployment of a recyclable covered metal stent was performed. **d** Contrast imaging confirmed complete closure with well-defined edges. APC, argon plasma coagulation.

**Fig. 4:**
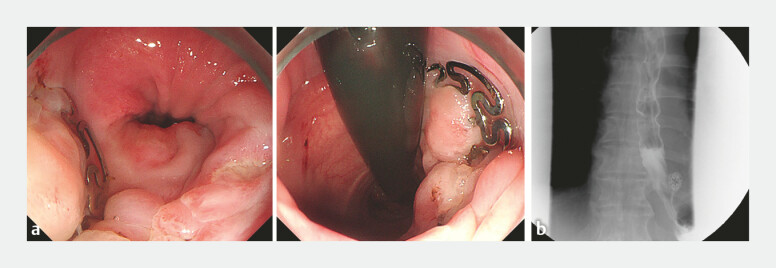
**a** Gastroscopy showed that the mucosa at the fistula site has completely healed when the stent was removed. **b** Contrast imaging confirmed the absence of leakage outside of the esophagus.

After 2 months, esophagomediastinal fistula in the lower esophagus, approximately 6mm in diameter ( Fig. 3 **a** ), was seen on gastroscopy when the stent was removed. The mucosal edges were treated with argon plasma coagulation (APC; Fig. 3 **b** ), and the fistula was successfully closed using a novel Over-The-Scope-Clip (OTSC, Star Clip) combined with endoscopic fully covered self-expanding metallic stent placement ( Fig. 3 **c** ; Video 1 ). On postoperative day 1, contrast imaging confirmed complete closure with well-defined edges ( Fig. 3 **d** ). At a 3-month follow-up, gastroscopy showed that the mucosa at the fistula site has completely healed when the stent was removed ( Fig. 4 **a** ). Contrast imaging confirmed the absence of leakage outside of the esophagus. ( Fig. 4 **b** ). After resuming oral feeding, the patient was discharged.

Endoscopic fully covered self-expanding metallic stent placement combined with a novel over the-scope clip for the closure of a complex esophagomediastinal fistula.Video 1

Management of an esophagomediastinal fistula remains challenging and is often associated with high morbidity and mortality 1 . Standard therapeutic approaches typically involve endoscopic and surgical interventions 2 , but the optimal protocol still needs to be determined. The successful management of an esophagomediastinal fistula requires prompt elimination of contamination in the mediastinum. The timely management of our patients resulted in satisfactory clinical outcomes on the healing of fistula as well as the improvement of feeding, suggesting that endoscopic fully covered self-expanding metallic stent placement combined with a novel over the-scope clip is effective and feasible. Our report offers practical evidence for similar cases.

Endoscopy_UCTN_Code_TTT_1AO_2AI
